# Image to English translation and comprehension: INT2-VQA method based on inter-modality and intra-modality collaborations

**DOI:** 10.1371/journal.pone.0290315

**Published:** 2023-08-30

**Authors:** Xianli Sheng

**Affiliations:** Institute of Foreign Languages and Tourism, Puyang Vocational and Technical College, Puyang, Henan, China; Kwame Nkrumah University of Science and Technology, GHANA

## Abstract

Existing visual question answering methods typically concentrate only on visual targets in images, ignoring the key textual content in the images, thereby limiting the depth and accuracy of image content comprehension. Inspired by this, we pay attention to the task of text-based visual question answering, address the performance bottleneck issue caused by over-fitting risk in existing self-attention-based models, and propose a scenario text visual question answering method called INT2-VQA that fuses knowledge manifestation based on inter-modality and intra-modality collaborations. Specifically, we model the complementary priori knowledge of locational collaboration between visual targets and textual targets across modalities and the contextual semantical collaboration among textual word targets within a modality. Based on this, a universal knowledge-reinforced attention module is designed to achieve a unified encoding manifestation of both relations. Extensive ablation experiments, contrast experiments, and visual analyses demonstrate the effectiveness of the proposed method and prove its superiority over the other state-of-the-art methods.

## 1. Introduction

**Fig 1:**
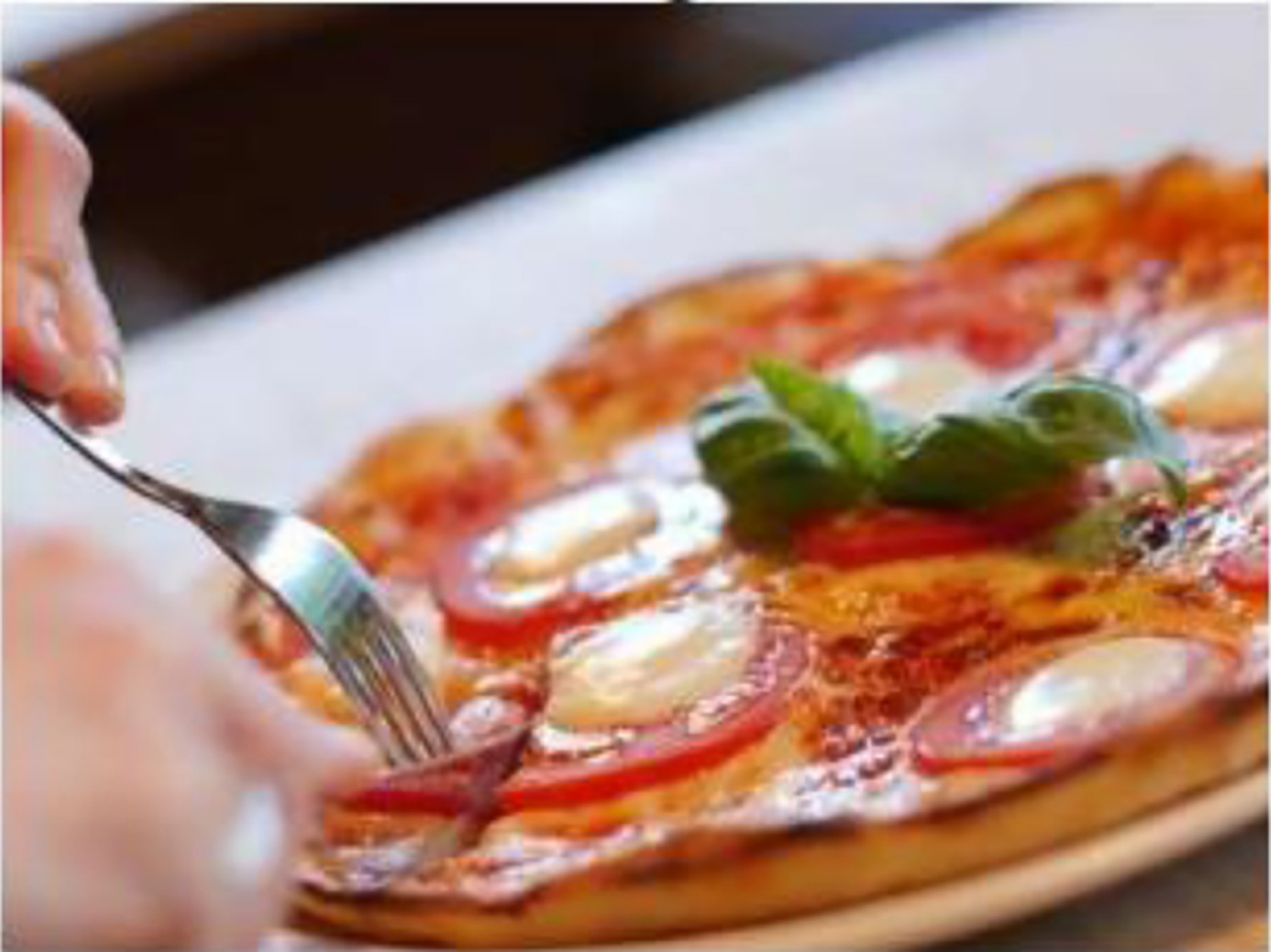
The general question answering task. Question: Which country is this food Originated from? Answering: Italy.

Visual Question Answering (VQA) is a typical task in the intersection of computer vision and natural language processing, and it has been a research hotspot in recent years. It takes an image and a question as input, aiming to design models that can fuse and reason over multi-modality inputs and generate natural language answers (usually in English) to the questions. Fig 1 illustrates an example of the general VQA task.

The models need to carefully comprehend the image and the question and perform reasoning to achieve accurate answer prediction, making it a highly challenging task. In recent years, significant progress has been made by researchers in the VQA task, achieving near-human-level accuracy on some commonly used VQA benchmark datasets [1]. However, most of these methods overlook the comprehension of scenario text, which is important information in the image, thus limiting the depth of research.

**Fig 2:**
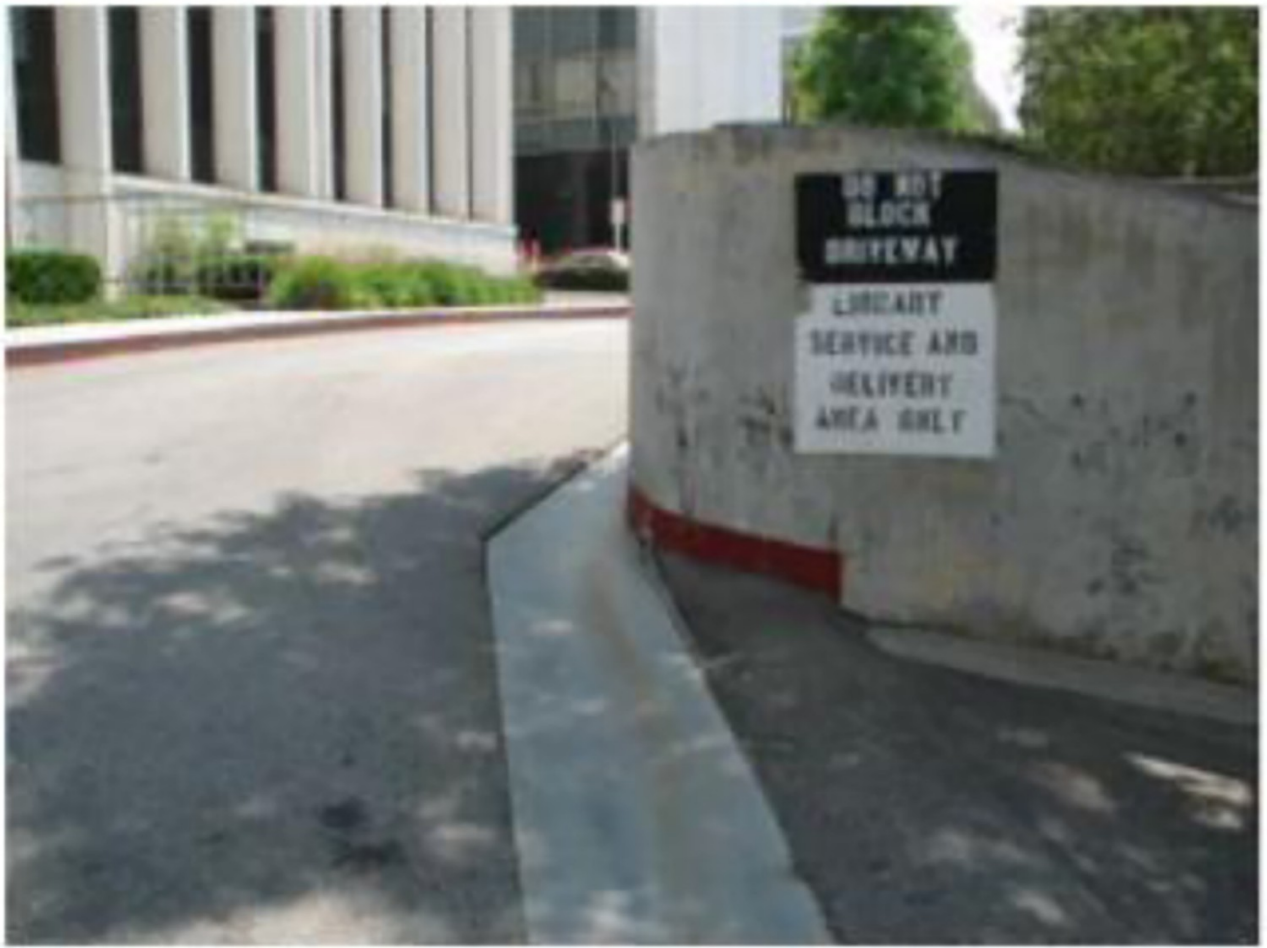
The scenario text question answering task. Question: What is written in the black rectangle on the top right? Answering: Do not block driveway.

Literature [2] and Literature [3] proposed integrating textual content into VQA, forming the task of TextVQA (Text Visual Question Answering) and ST-VQA (Scenario Text Visual Question Answering) respectively, along with the construction of benchmark datasets. Fig 2 illustrates an example of the ST-VQA task, where the questions are related to the scenario text in the image, requiring the model to establish unified collaborations between the question, visual targets, and scenario text to generate correct answers.

To understand the scenario text in the image, ST-VQA models typically incorporate an optical character recognition (OCR) system to detect and recognize text targets in the image. Several methods have been proposed based on the extracted OCR targets [4, 5]. LoRRA (look read reason and answer) method is an extension of Pythia [6], which is originally designed for traditional VQA model. LoRRA incorporates an existing OCR system to detect textual targets in images and adds an additional OCR attention branch for scenario text encoding. It performs inference by reasoning on words from a fixed vocabulary and words recognized by OCR, selecting the word with the highest probability as the answer. However, LoRRA fails to build rich OCR target characteristics, thus limiting its comprehension of the information contained in OCR targets. Additionally, its shallow attention fusion model cannot perform deep reasoning. To address these issues, literature [4] proposed the M4C-VQA (multi-modality multi-copy mesh) model, which is a powerful baseline method for the ST-VQA task. It leverages a multi-modality Transformer structure to fuse characteristics from different modalities into a shared space. In this space, the all collaborations are learned automatically by self-attention models. M4C-VQA treats ST-VQA as a sequence generation task and combines it with a DPN (dynamic pointer network) module to achieve precise answer generation. However, the self-attention layer in the Transformer of M4C-VQA is fully connected, distributing attention over the entire context, neglecting the importance of local context around specific targets or text. Besides, M4C does not consider the relative locational relations between targets in the image, leading to sub-optimal performance in questions involving locational relations.

In the ST-VQA task, some questions involve reasoning about the relative locational relations between targets. For example, the position representation, e.g., the top right, in Fig 2. To address such questions, the SA-M4C-VQA (Spatially Aware-M4C) method incorporates 12 predefined locational relations to establish collaborations between visual targets and OCR targets, capturing enhanced knowledge of relative locational relations [7]. This information is integrated into each attention layer of the Transformer, improving the performance of the original M4C-VQA method. However, the locational quantization strategy that manually defined relations may not accurately represent the locational collaboration for closely related targets. In other words, the predefined modeling method lacks fine-grained expression of locational relations, making it difficult to differentiate targets in close proximity.

In order to deal with the above problems in existing models, this paper proposes an Inter-Modality and Intra-Modality Visual Question Answering method, i.e., INT2-VQA, which is based on the collaborations between modalities and within modalities. By modeling the complementary priori knowledge of the intra-modality locational collaboration and the inter-modality contextual semantical collaboration, the proposed method enhances the model’s comprehension of complex scenarios. The intra-modality locational collaboration encodes the relative positions between visual targets and OCR targets, accurately capturing fine-grained positional relations between pairs of targets. The inter-modality contextual semantical collaboration describes the semantical similarity between words corresponding to OCR targets and predicted answer words, encoding the words with contextual semantical collaborations to improve the accuracy and reliability of the answer generation process. To evaluate the effectiveness of the proposed INT2-VQA method, extensive experiments are conducted on the aforementioned two publicly available datasets. The experimental results show significant performance improvements compared to the current other benchmark methods on both datasets.

In summary, the main contributions of this paper are as follows:

1. We propose an INT2-VQA method based on inter-modality and intra-modality collaborations, which incorporates reinforced knowledge for richer information manifestation;
2. We model both the locational collaboration between visual targets and the contextual semantical collaboration between words, guiding the model to accurately locate key targets and text entities;
3. We have thoroughly divided VQA into six classes, and introduced them from their reasons, details, and shortcomings.
4. Extensive contrast and ablation experiments are conducted on different open-source datasets. The experimental results demonstrate that the INT2-VQA method outperforms existing state-of-the-art (SOTA) methods.

The structure of this paper is organized as follows. The first section is the introduction, followed by the second section on related work. The third section shows the proposed method, while the fourth section describes the extensive experiments conducted. The fifth section is the discuss, and finally, the sixth section provides a summary of the findings.

## 2. Related works

**Fig 3:**
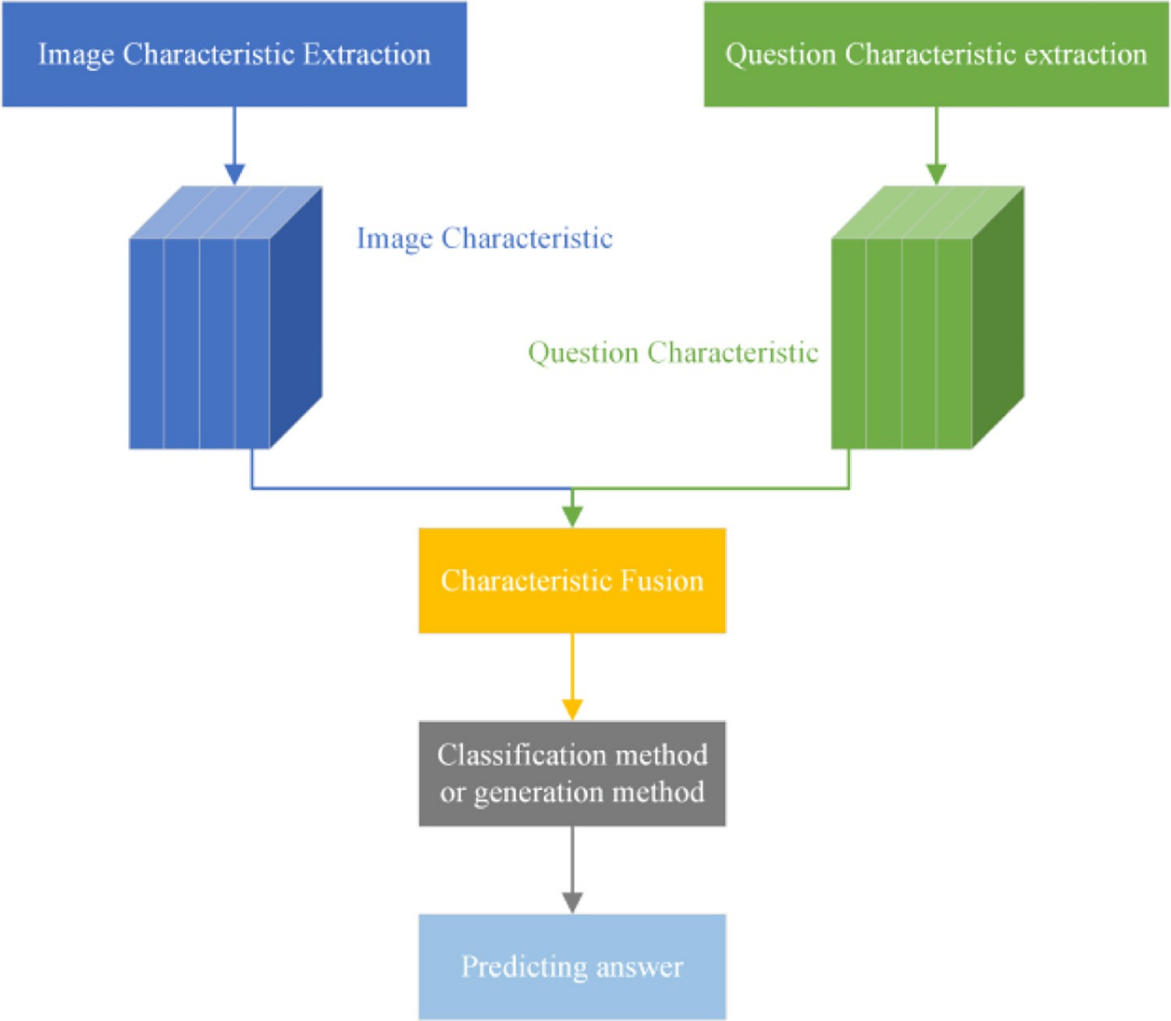
The general architecture of VQA model.

Since the emergence of the VQA task, researchers have proposed various high-performing models. Most of these models can be summarized into a framework consisting of four components: image characteristic extraction module, question characteristic extraction module, characteristic fusion module, and answer generation module. The general framework of the VQA model is illustrated in Fig 3.

The image characteristic extraction module mainly utilizes VGG-Net [8], ResNet [9], and GoogleNet [10] to extract image characteristics. With the continuous development of target detection, the use of Faster R-CNN [11] for image characteristic extraction has become mainstream.

The question characteristic extraction module primarily employs language encoding models such as LSTM (Long Short-Term Memory) [12], GRU (Gate Recurrent Unit) [13], Transformer [14], and BERT (Bidirectional Encoder Representation from Transformers) [15] to extract question characteristics.

The role of the characteristic fusion module is to map image characteristics and text characteristics to the same characteristic space and perform interactive fusion. This module is the core part of the VQA model.

The answer generation module can employ classification or generation methods. For multiple-choice tasks, the fused characteristics are fed into a classifier to obtain the probability scores for each candidate answer, and the answer with the highest score is selected as the correct answer. For open-ended tasks, the fused characteristics are fed into an RNN (Recurrent Neural Network) or LSTM model to generate the answer.

In this section, the models are categorized into six classes based on their peculiarities, i.e., joint embedding-based methods, attention-based methods, scenario reasoning-based methods, external knowledge-based methods, contrastive learning-based methods, and 3D point cloud-based methods. For each of these six classes, the reasons for the model’s proposal, the model’s ideas, the connections between models, and the problems existing in the models are described detailedly in this section.

### 2.1 Joint embedding-based methods

In order to accomplish the VQA task, researchers initially explored the concept of joint embedding of images and text. Joint embedding methods often integrate the two types of characteristics through operations such as concatenation, element-wise multiplication, or element-wise addition.

One of the earliest studies on joint embedding methods in VQA models was Neural-Image-QA proposed by [16]. They combined the latest advancements in image representation and natural language processing to address challenging tasks in image questioning. The model incorporated CNN (Convolutional Neural Network) and LSTM into an end-to-end architecture to predict answers to questions based on images. The Neural-Image-QA model generated answers in a generative manner. On the contrary, literature [17] proposed the mQA model, which treated the VQA task as a classification task and fed the characteristic vectors into a linear classifier to generate answers from a predefined vocabulary. Inspired by the deep residual structure, literature [18] introduced Multi-modality Residual Networks (MRN) for multi-modality residual learning in VQA task. MRN extended the idea of deep residual learning. Unlike deep residual learning, MRN effectively learned joint representations from visual and language information by employing element-wise multiplication for joint residual mapping. In contrast to most models that utilize separate image encoders and question encoders, the model proposed by [19] consisted of three CNNs: an image CNN for encoding image content, a sentence CNN for encoding questions, and a multi-modality convolutional layer to learn their joint representation for spatial classification among candidate answer words.

Joint embedding-based methods simply concatenate image characteristics and text characteristics without establishing correspondences between image regions and question keywords. These methods directly utilize all visual and text information to derive answers. However, there is a significant portion of irrelevant information present in both visual and text characteristics, which can interfere with the final answer classification or generation. This fusion method is relatively rough and lacks reasoning based on the question, leaving room for improvement.

### 2.2 Attention-based methods

The attention mechanism in deep learning mimics the human attention mechanism. Humans quickly scan images or text, focusing on important regions and keywords to rapidly grasp the main information. The attention mechanism has been rapidly adopted in various fields of artificial intelligence and has achieved promising performance. In the context of VQA tasks, attention methods enhance the model’s comprehension of both visual and semantical information. In recent years, attention methods can be broadly categorized into question-guided attention, co-attention, and multi-granularity attention methods. The following will primarily focus on introducing these three categories of methods.

#### 2.2.1 Question-guided attention method

Early attention methods utilized the question to compute the importance of different regions in an image and identify regions closely related to the question. For example, literature [20] learned to answer visual questions by selecting image regions that are relevant to text-based queries. They simply multiplied visual characteristics by attention weights obtained from the product of visual and textual characteristics, and updated the visual characteristics with the multiplied results. Literature [21] employed a ResNet-based model to extract image characteristics, while the input question was fed into a multi-layer LSTM. Then, using the concatenated image characteristics and the final state of the LSTM, attention distributions over the image characteristics were computed. Existing methods only computed visual attention distributions once. To further enhance the fusion of cross-modal characteristics, literature [22] proposed the Stacked Attention Network (SAN). This model consists of a multi-step reasoning process and establishes multiple layers of attention mechanisms. It performs multiple queries on an image based on the semantics of the question to gradually infer the answer.

#### 2.2.2 Co-attention method

Co-attention method further improves the early attention mechanisms. Specifically, co-attention method considers not only the attention of obtaining image characteristics guided by the question but also the attention of using image characteristics to obtain question information. To enhance the performance of models in VQA, a fine-grained comprehension of both visual and textual content is required. Specifically, it is challenging to establish a collaboration between the keywords in the question and the key targets in the image. Literature [23] proposed the Deep Modular Co-Attention Networks, which borrowed the framework of the Transformer model. The encoder consists of six stacked self-attention units primarily used for textual interactions, while the decoder combines self-attention units and guided attention units to enable interaction between textual and visual characteristics. There have been several improvements based on the Transformer structure. Literature [24] introduced an improved attention-based network structure. They added the Attention over Attention (AoA) module to the encoder-decoder framework, which determines the relation between attention results and queries and generates weighted averages for each query in the attention module. Additionally, they proposed a multi-modality fusion module that combines visual and textual information, dynamically determining the amount of visual and textual information to consider. In VQA tasks, multi-modality predictions often require understanding visual information from a macro to micro level. Therefore, dynamically modulating global and local dependencies in the Transformer becomes a new challenge. Literature [25] proposed a TRansformers with Adaptive Routing (TRAR) mechanism to address this issue. In TRAR, each visualizable Transformer layer is equipped with a routing module with varying attention widths. The model can dynamically select the appropriate attention range for each instance based on the output of the previous reasoning step, thus determining the optimal routing path for each instance. Methods based on the Transformer structure have achieved significant success in visual question answering. However, these models often have deeper networks and wider embedding dimensions, making it challenging to deploy them on resource-constrained platforms. Designing VQA models that support runtime adaptive pruning is a valuable task to meet efficiency constraints on different platforms. Literature [26] proposed the Dual-Stage Lightweight Transformer (DST), a universal framework that seamlessly integrates with any Transformer-based VQA model. DST simplifies the model in terms of width and depth, trains a single model at a time, and obtains multiple efficient sub-models adaptable to different platforms. Furthermore, literature [27] introduced a cross-modality attention distillation framework to train dual-encoder models for visual language comprehension tasks. The framework employs attention distributions from the fusion encoder model for both image-text and text-image modalities to guide the training of the dual-encoder model.

#### 2.2.3 Multi-granularity attention method

Existing visual language models either employ fine-grained target-centric image-text alignment or coarse-grained holistic image-text alignment. While both approaches have been effective, they still have some limitations. Fine-grained detection can identify all possible targets in an image, but some of these targets may be irrelevant to the text. Target-centric characteristics do not easily capture the relations between multiple targets. On the other hand, coarse-grained methods struggle to effectively learn fine-grained alignment between vision and language. Literature [28] proposed a novel method called X-VLM for multi-granularity visual language pretraining. They reconstructed existing datasets into visual concepts and their corresponding texts. Visual concepts could be targets, regions, or the image itself. The model aligns text with relevant visual concepts using a multi-granularity approach. Due to the high diversity of image characteristics, the lack of language structure and grammar rules, language characteristic s are prone to losing detailed information. To better learn attention between vision and text, literature [1] introduced a new multi-granularity alignment architecture that jointly learns the correlations at three different levels: concept-entity level, region-noun phrase level, and spatial-sentence level. They constructed a decision fusion module to combine the outputs of Transformer modules at different granularities. To simultaneously pretrain the encoder for multi-modality representation extraction and the language decoder for sentence generation, literature [29] proposed a pretrained universal encoder-decoder network (Uni-EDEN) to facilitate visual language perception and generation. The model undergoes pretraining using multi-granularity visual language proxy tasks: Masked Object Classification (MOC), Masked Region Phrase Generation (MRPG), Image-Sentence Matching (ISM), and Masked Sentence Generation (MSG). The multi-granularity visual language proxy tasks aim to align visual content with language representations at different granularities, ranging from individual labels and phrases to natural sentences.

Attention-based methods have been the mainstream approach in VQA tasks and have received significant attention from researchers. Models based on attention methods have been continuously improved and achieved outstanding performance. However, attention methods mainly focus on image regions and textual keywords, without capturing the relations between targets in the image, providing little help for reasoning-based questions. Further exploration is needed to integrate reasoning chains into VQA tasks and accurately locate image regions relevant to the answers.

### 2.3 Scenario reasoning-based methods

Scenario graphs are structured representations of scenarios that can clearly express targets, attributes, and relationships between targets in a scenario [30]. Currently, people no longer settle for simply detecting and recognizing targets in images but require higher-level visual comprehension and reasoning tasks to capture the relations between targets in a scenario. scenario graphs serve as powerful tools for understanding scenarios, and as a result, they have garnered significant attention from researchers. Relevant research in this area is often multi-modal, complex, and rapidly evolving.

The main idea behind scenario graph-based VQA models is to utilize semantical cues from questions to guide visual content reasoning. Literature [31] proposed a new approach called scenario Graph Convolutional Networks (scenarioGCN) that uses pre-trained detectors and visual relation encoders to vectorize targets and relationships in the scenario graph. It then applies scenario graph convolution, which utilizes the information of targets and relationships to update the hidden states of each node. In contrast to the scenarioGCN model, literature [32] introduced a language-guided graph neural network framework called GraphVQA. This framework first transforms the question into M instruction vectors and then performs message passing on each instruction vector using a graph neural network. Finally, it aggregates the final states after message passing and predicts the answer. In previous studies, images could be appropriately represented through scenario graphs, but questions were often simply embedded, failing to represent the complete semantics well. To address this, literature [33] proposed a Graph Matching Attention (GMA) network that constructs the scenario graph of an image not only from target appearance, geometric features, and spatial relationships but also utilizes syntax trees and language features extracted from the question to construct the graph. It first obtains intra-modality relationships through a bi-stage graph encoder, and then infers relations between images and questions using bidirectional cross-modality graph matching attention and propagates cross-modality information.

Existing VQA models incorporate various pieces of information but lack fine-grained answer searching, which may introduce additional noisy data and interfere with the model’s ability to provide correct answers. Compared to traditional VQA methods, scenario graphs can capture the basic information of an image in the form of a graph structure, making scenario graph-based VQA methods superior to traditional algorithms. However, visual question answering algorithms based on scenario graphs still have room for improvement. While the model can search for answers in the scenario graph based on the question, its performance is not ideal for problems involving counting, reasoning, time, and other aspects. Additionally, the process of reasoning and inferring answers in scenario graph-based visual question answering is not transparent enough, and further research is needed in terms of interpretability.

### 2.4 External knowledge-based methods

VQA tasks typically involve complex and diverse questions that cannot be answered solely based on limited visual information. In such cases, visual question answering models need to obtain information from external knowledge bases as supporting evidence for answering questions. For example, for a question like which object in this image can be used to protect the head, it is necessary to comprehend that protecting the head is a function and then search for the target that serves this purpose. VQA methods based on external knowledge are a future research trend, especially in specific professional fields where they have certain application value.

#### 2.4.1 Knowledge reasoning methods

Literature [34] proposed a VQA method called Ahab that performs reasoning on image content in large-scale knowledge bases. Ahab first detects relevant content in the image and associates it with available information in the knowledge base. It processes natural language questions into appropriate queries and combines the image and knowledge base information for querying. This querying may require multiple reasoning steps to complete, and the final answer is formed based on the feedback from the query. Previous methods could only ask a specific set of questions to the system, and the query generation methods used required very specific question formats. To be applicable to general knowledge bases and answer a wide range of questions, literature [35] proposed a visual question answering method that constructs textual representations of the semantical content of images and combines them with textual information from the knowledge base to deepen the comprehension of the observed scenario, enabling the model to answer more extensive and complex questions than before. The existing methods rely on retrieving basic facts for question answering, but in real-world applications, questions may be posed that involve facts not present in the knowledge graph. Literature [36] developed a new question answering framework called FVQA that can handle the FVQA task even when the required edges are missing from the knowledge graph. In this process, the method combines complementary lexical characteristics and knowledge graph semantical characteristics, improving the accuracy of answer retrieval. Knowledge reasoning methods have achieved good results in complex problems that require external knowledge, but the methods based on knowledge reasoning cannot adaptively select specific domain knowledge from large-scale knowledge graphs, and the reasoning process is relatively simple. In future work, it is worth further studying models that can select knowledge within a small range from knowledge graphs to perform multi-hop and more complex reasoning.

#### 2.4.2 Knowledge search methods

Recent research has begun to focus on how to integrate knowledge search methods into VQA. These methods study the integration of knowledge bases and retrieval methods with VQA datasets and provide a set of relevant facts for each question. Literature [37] proposed the knowledge-based baseline ArticleNet for VQA. Firstly, all possible queries for each question are collected by combining the words in the question with the words recognized by the trained image and scenario classifier. Then, the most popular articles are obtained for each query using the Wikipedia search API. Finally, the most relevant sentence is selected from the articles based on the occurrence frequency of these query words in the sentence to find the answer to the question by selecting the highest-scoring word from the retrieved sentence.

The VQA based on knowledge search has attracted increasing attention from researchers. However, most questions require only a small amount of knowledge from the knowledge base. Overcoming the challenge of excluding noisy information and accurately extracting relevant knowledge is necessary.

### 2.5 Contrastive learning-based methods

Self-supervised learning is a type of unsupervised learning paradigm that does not require manually annotated class label information. Instead, it utilizes the supervisory information provided by the data itself to learn the feature representations of sample data, which can be used for downstream tasks. Contrastive learning is an important method in self-supervised learning. In visual language representation learning, image-text alignment is achieved through contrastive learning. This alignment strategy can achieve success because it maximizes the mutual information (MI) between images and matching texts. Mutual information is a method for measuring the mutual dependency between variables, and it measures the relation between images and questions by distinguishing positive sample pairs from negative sample pairs.

Learning the interaction between image and text through multi-modality encoders is challenging. To address this issue, literature [38] proposed the ALBEF model, which introduces image-text contrastive learning. It uses image encoders, text encoders, and multi-modality encoders for pre-training. The pre-training objective is to maximize the mutual information of image-text pairs, fine-grained interaction between image and text, and image-text pairing. Literature [39] proposed a unified visual language pre-training model called VLMo, which jointly learns a dual encoder and a fusion encoder shared with the MoME Transformer network. MoME introduces a modality expert pool to encode modality-specific information and uses a shared self-attention module to align different modalities. Through unified pre-training with MoME, model parameters are shared in image-text contrastive learning, masked language modeling, and image-text matching tasks. The encoders of most models primarily extract information from irrelevant/noisy image patches or text tokens. To address this, literature [40] proposed a new visual language pre-training framework called TCL. Unlike previous research that simply aligns image and text representations through cross-modal contrastive loss, TCL further considers modality-specific supervision, which in turn benefits cross-modal alignment and joint multi-modality embedding learning. To incorporate local information and structural information into representation learning, TCL further introduces local mutual information, maximizing the mutual information between global representations and local information of image patches or text tokens.

Contrastive learning aims to bring matched image-text pairs as close as possible while keeping unmatched image-text pairs far apart. The goal of contrastive learning is to make the fusion encoder more capable of learning multi-modality interactions. However, contrastive learning in visual question answering still has certain limitations. It enhances the global mutual information between images and texts while neglecting local information and structural information in the input. Additionally, certain noises may dominate the MI, leading the model to learn irrelevant features.

### 2.6 3D point cloud-based methods

VQA has made significant progress in recent years. However, current research primarily focuses on the task of 2D image-based question answering. Researchers have been exploring the expansion of VQA into the 3D domain, which can facilitate artificial intelligence’s perception of 3D real-world scenarios, enabling the simulation of realistic scenarios and widespread applications. Unlike image-based VQA, 3D question answering takes point clouds as input and requires both language processing and 3D scenario comprehension to answer questions related to the 3D scenario.

Existing models based on 2D images face challenges in accurately comprehend the 3D world. For example, 2D images lack accurate perception of relative directions and distances in 3D scenarios, and targets can be occluded when overlapping. To address these issues, literature [41] proposed a baseline model for 3D question answering called ScanQA. The ScanQA model includes 3D and language encoders, a 3D and language fusion module, target localization, and a QA layer. The 3D and language encoder layer converts the question into a feature vector representation and transforms the point cloud into characteristic candidate boxes. The 3D and language fusion layer combines multiple 3D target characteristics guided by a Transformer-based encoder and decoder layer along with textual information. The target localization and QA layer evaluate the target boxes and target labels and predict answers related to the question and scenario content. Compared to other 3D scenario understanding tasks, 3D question answering requires a higher level of 3D geometric understanding. It not only needs to understand the appearance and geometric structure of objects but also spatial relations between different targets. Literature [42] introduced a novel Transformer-based 3D question answering framework called 3DQA-TR. It utilizes a language tokenizer for question embedding, extracts appearance and geometric information using two encoders, and then associates multi-modality information of appearance, geometry, and language questions using 3D-LBERT to predict the target answer. Traditional 3D scenario understanding tasks mostly focus on individual targets and overlook target relations. Literature [43] introduced visual question answering in real 3D scenarios, aiming to answer all possible questions given a 3D scenario. They designed TransVQA3D, which first uses a cross-modal Transformer to fuse question and target characteristics. Then, by initializing the scenario graph using the scenario graph embedding and attending to the scenario graph using additional edges, it captures relations between targets and infers answers.

3D scenario comprehension is a relatively emerging research field. Compared to reasoning based on 2D images, performing reasoning in real 3D scenarios can avoid spatial ambiguity in 2D data, thus obtaining real geometric information and target relations. Additionally, 3D scenarios usually involve more targets and complex target relations. Although researchers have made significant efforts in exploring spatial representations to enhance scenario comprehension, there are still shortcomings in 3D perception (such as counting, verification, and existence) and acquiring target attributes (such as size, texture, and structure), which provide further room for improvement.

## 3. Materials and methods

In the context of ST-VQA tasks, this paper proposes improvements based on the M4C-VQA model. By jointly modeling two complementary priori knowledge sources, namely the locational relation between modalities and the contextual semantic relation within modalities, the proposed INT2-VQA model is developed to enhance knowledge manifestation.

### 3.1 M4C-VQA model overview

The M4C-VQA model is designed to handle both question and image modalities as input. After unifying the multi-modality data representation, it utilizes a multi-layer Transformer network to deeply integrate the multi-modality information. Finally, an iterative answer prediction is performed using a DPN.

#### 3.1.1 Unified characterization of multi-modality data

**Fig 4:**
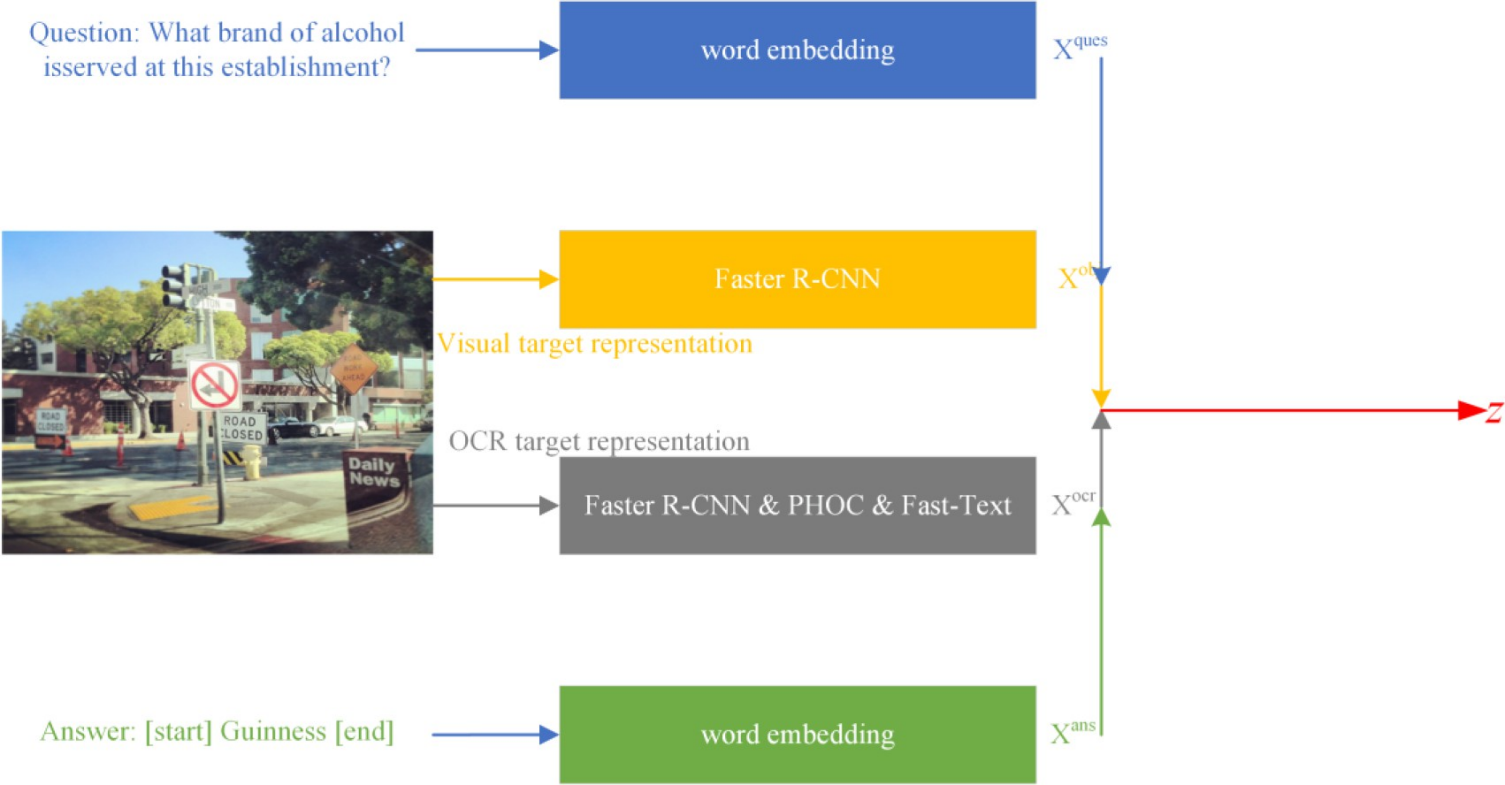
Architecture of the M4C-VQA model.

As shown in Fig 4, the M4C-VQA model performs word embedding for the words in the question and answer. For the image, it extracts visual target regions and OCR target regions. Visual target representation and OCR target representation are then generated for the extracted regions.

For a given question consisting of up to *K* words, each word is mapped to a *d*-dimensional characteristic vector using a pretrained BERT-base model. The representations of each word are concatenated to obtain the question characteristic *X*^*ques*^∈**R**^*K*×*d*^.

For an input image, a pretrained Faster R-CNN is used to extract up to *M* visual target regions. Visual characteristic $x_{m}^{vis}$ and coordinate characteristic $x_{m}^{box}$ are extracted for the *m*-th visual target (where *m* ∈ {1,…,*M*}). Linear mapping is applied to unify these characteristics and map them to a *d*-dimensional space, resulting in the visual target representation $x_{m}^{obj}$. Finally, the *M* visual target representations are element-wise concatenated to obtain *X*^*obj*^∈**R**^*M*×*d*^.

For text in the image, an external OCR system is used to extract up to *N* text target regions and obtain their corresponding word representations. For the *n*-th text target (where *n* ∈ {1,…,*N*}), four types of characteristics are extracted: word vector characteristics based on the FastText method [44], character characteristics based on the PHOC (Pyramidal Histogram of Characters) method [45], visual characteristics based on Faster R-CNN, and bounding box coordinate characteristics of the text regions. Linear mapping is then applied to unify these four characteristics and map them to a *d*-dimensional space, resulting in the representation of each text target $x_{n}^{ocr}$. Finally, the *N* text target representations are concatenated to obtain *X*^*ocr*^∈**R**^*N*×*d*^.

For an answer sequence consisting of up to *T* words, each word is mapped to a *d*-dimensional vector $x_{t}^{ans}$ (where *t* ∈ {1,…,*T*}). The representations of the T words are concatenated to obtain *X*^*ans*^∈**R**^*T*×*d*^. In the answer vocabulary, two special words, *[start]* and *[end]*, are added to denote the start and end symbols in the answer generation process.

The four sets of multi-modality characteristics, *X*^*ques*^, *X*^*obj*^, *X*^*ocr*^, and *X*^*ans*^, are concatenated to obtain the fused characteristic *Z*∈**R**^(*K*+*M*+*N*+*T*)×*d*^. This fused characteristic is then input to the Transformer encoder model [46] at the encoding layer for deep fusion of the characteristics.

#### 3.1.2 Auto-regressive answer prediction

Since the answer may consist of a combination of OCR words and vocabulary words, the M4C-VQA model introduces an auto-regressive answer prediction module that combines a DPN. Adaptive selection is performed between OCR words and the predefined answer vocabulary to generate the predicted output at time step *t*. The prediction at each step is then used as input for the next decoding iteration until the termination symbol is output.

Let $z_{t}^{ans}$ represent the output characteristic of the Transformer encoder for the answer word at time step *t*. The predefined answer vocabulary consists of *V* words. $z_{t}^{ans}$ is passed through a linear layer to obtain a *V*-dimensional vocabulary word score vector $y_{t}^{voc}\in R^{v}$. Additionally, $\left\{z_{1}^{ocr},z_{2}^{ocr},\ldots ,z_{N}^{ocr}\right\}$ represents the characteristics obtained by applying the Transformer to *N* OCR words. The DPN module aims to compute the relevance scores between $z_{t}^{ans}$ and any OCR word, as shown in (1):

$$y_{t,n}^{ocr}=line_{d}(z_{t}^{ans})line_{d}(z_{n}^{ocr})$$ (1)

In which, line(.) represents a linear mapping layer that outputs a *d*-dimensional vector. $y_{t,n}^{ocr}$ represents the score between the answer word at time step *t* and the *n*-th OCR word. By computing this score for all OCR words, an OCR score vector $y_{t}^{ocr}\in R^{N}$ is obtained.

Finally, the prediction vector is obtained by concatenating the two aforementioned vectors. The cumulative BCE (Binary Bross-Entropy) loss between the predicted answer and the ground truth answer is calculated to achieve end-to-end optimization of the entire M4C-VQA model.

### 3.2 Proposed INT2-VQA model

Thanks to the powerful modeling capabilities of the multi-layer Transformer model, the M4C-VQA model can learn fine-grained semantical correlations between different modalities. However, it has two weaknesses:

1. Although each visual target and OCR target contains spatial positional information, the fusion of this spatial characteristic with other types of characteristic loses explicit coordinate meaning, making it difficult for the M4C-VQA model to accurately comprehend the locational relations between targets.
2. When predicting answers, the output words need to be selected from OCR target words and vocabulary words. The semantical collaboration between these words from different sources are not explicitly modeled in M4C-VQA, making it difficult to accurately comprehend the semantical collaboration between words from multiple sources during the answer prediction process.

**Fig 5:**
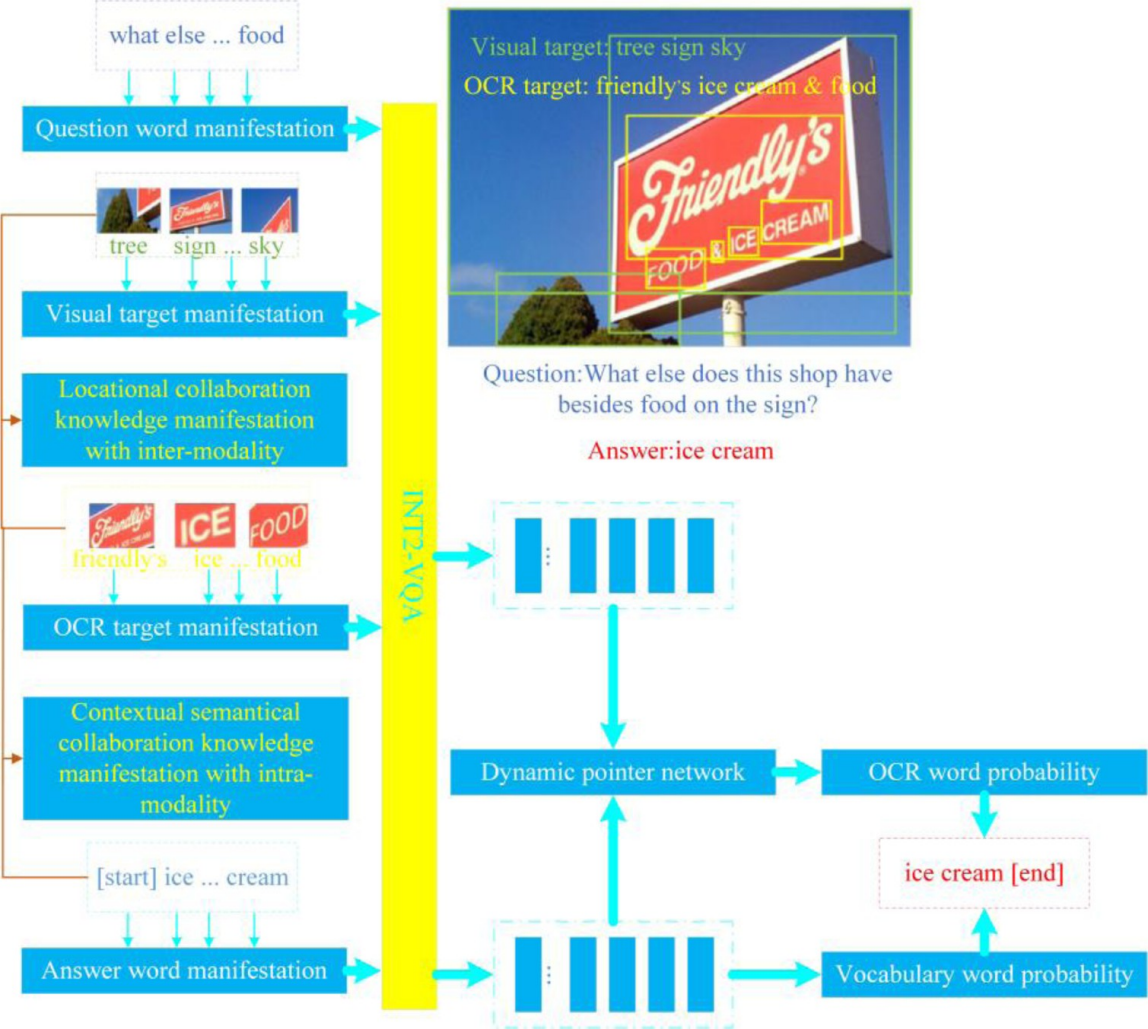
Architecture of the proposed INT2-VQA model.

To address these issues, this paper extracts locational collaboration knowledge representations with inter-modality, i.e., between visual targets and OCR targets in the image modalities, and contextual semantical collaboration knowledge manifestations with intra-modality, i.e., between OCR targets’ extracted words and predicted words. Based on the M4C-VQA architecture, an improved method called INT2-VQA is proposed to encode and represent these two types of collaboration knowledge. The overall framework of INT2-VQA is illustrated in Fig 5, where the locational collaboration between visual targets and OCR targets and the contextual semantical correlation between OCR words and predicted answer words contribute to the improved accuracy of ST-VQA.

#### 3.2.1 Locational collaboration knowledge manifestation with inter-modality

**Fig 6:**
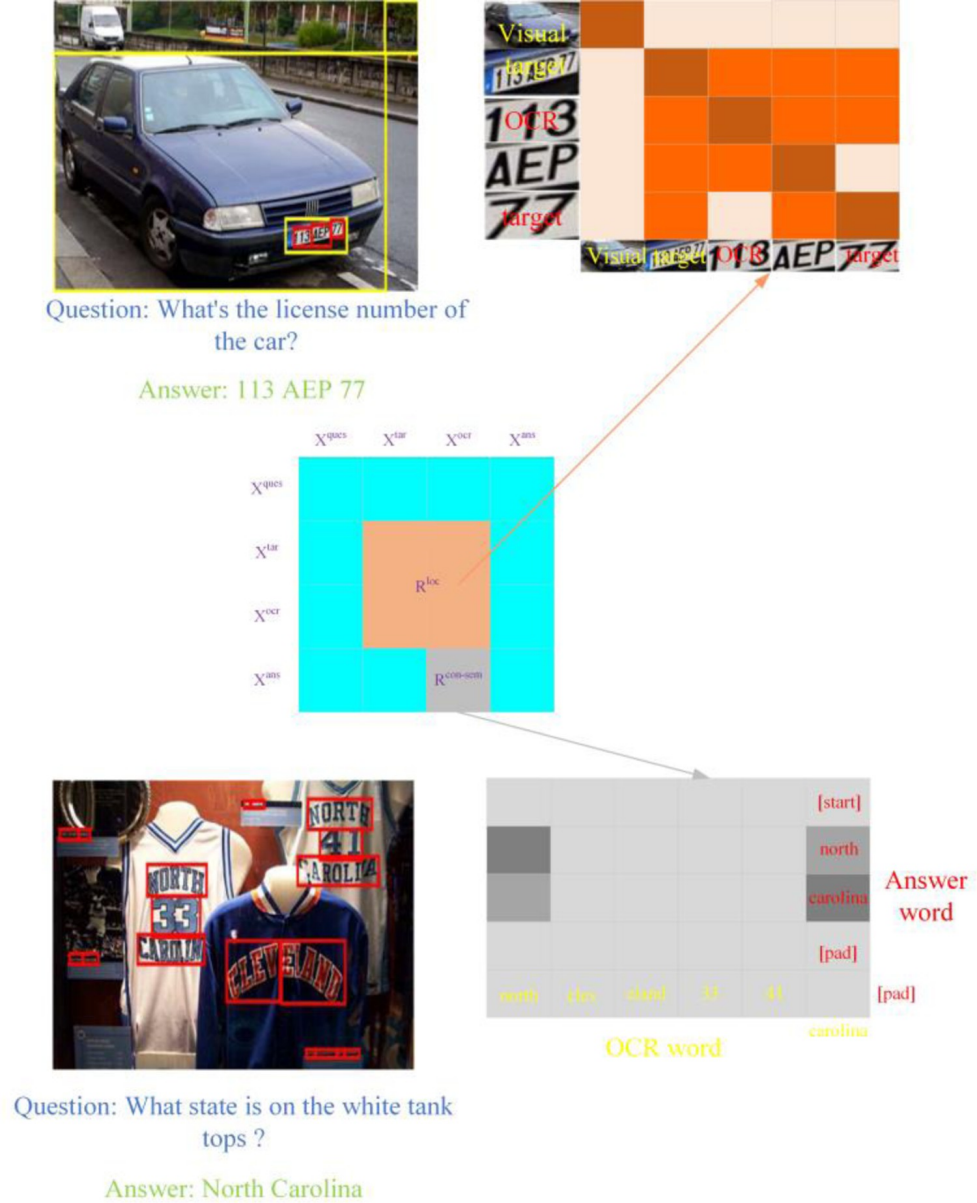
The sketch map of locational and contextual semantical collaboration knowledge representation with inter-modality and intra-modality.

In the ST-VQA task, the model needs to comprehend the locational relations between visual targets and OCR targets in the image and reason based on that. As shown in Fig 6, given the question *what’s the license number of the car*?, the model first needs to detect the visual target corresponding to the license plate in the image, and then infer the relation between the numbers and the license plate, determining that only the digits inside the license plate target are the number to be answered.

Given the extracted characteristics of *M* visual targets, *X*^*tar*^, and *N* OCR targets, *X*^*ocr*^, they are considered as an target set consisting of *M+N* elements. The relative locational relations between any pair of elements are encoded to obtain locational collaboration knowledge representation. Specifically, given the bounding box information of any two targets, existing methods such as SA-M4C-VQA propose a spatial locational encoding approach based on a spatial relation graph, expressing spatial relative positions between any two targets using 12 predefined common locational relations (e.g., above, below, left, right, inside, outside, contains, and overlaps). However, this method does not provide fine-grained granularity in expressing spatial relation and may struggle to differentiate between closely locational targets. To address this issue, this paper adopts a coordinate-based relative locational encoding method.

Let the bounding box of the *i*-th target be represented as (*x*_*i*_, *y*_*i*_, *w*_*i*_, *h*_*i*_), where *x*_*i*_ and *y*_*i*_ represent the center coordinates of the bounding box, and *w*_*i*_ and *h*_*i*_ represent the width and height of the bounding box, respectively. The locational relation characteristic between the *i*-th and *j*-th targets is denoted as $x_{i,j}^{loc}\in R^{4}$, as shown in (2).


$$x_{i,j}^{loc}=[\log\frac{\left|x_{i}-x_{j}\right|}{w_{i}},\log\frac{\left|y_{i}-y_{j}\right|}{h_{i}},\log\frac{w_{j}}{w_{i}},\log\frac{h_{j}}{h_{i}}]$$ (2)

For the *M+N* targets, the above method is used to obtain the corresponding locational location manifestation *X*^*loc*^∈**R**^(*M*+*N*)×(*M*+*N*)×4^. Furthermore, *X*^*loc*^ is passed through a two-layer fully connected network to obtain the final locational relation knowledge manifestation *R*^*loc*^∈**R**^(*M*+*N*)×(*M*+*N*)^, as shown in (3).


$$R^{loc}=relu->line_{1}->relu->line_{d}(X^{loc})$$ (3)

In which, *relu* represents the ReLU layer, -> denotes the sequential connection between two layers.

#### 3.2.2 Contextual semantical collaboration knowledge manifestation with intra-modality

Some questions may involve combinations of words that are not spatially adjacent, such as *North* and *Carolina* shown in Fig 6. How to uncover the implicit contextual semantical relations between these words directly affects the accuracy of the final predictions. Additionally, since the answer consists of words from OCR and a fixed vocabulary and is predicted by two independent classifiers, the model needs to learn to comprehend the semantical correlations between words from these two different sources.

To address the above issues, we build contextual semantical collaboration knowledge between the words corresponding to OCR targets and the predicted answer words. We use the similarity between pre-trained GloVe (Global Vectors for Word Representation) word embeddings [47] to calculate the relative semantical relations between words. Specifically, given the word corresponding to the *i*-th OCR target and the *j*-th word of the predicted answer, represented by GloVe word vectors *e*_*i*_ and *e*_*j*_, respectively, their relative contextual semantical relation is calculated using the cosine similarity between the two word vectors:

$$x_{i,j}^{con-sem}=f_{\cos-sim}(e_{i},e_{j})$$ (4)

By calculating all the answer words and OCR words, given *N* OCR targets and an answer length of *T*, we obtain the contextual semantical correlation knowledge representation *R*^*con−sem*^∈**R**^*T*×*N*^. For answers with a length shorter than *T*, a special word *[pad]* is used to represent the padding. The similarity between the special words *[start]*, *[end]*, and *[pad]* and the OCR words is not calculated, and their values are set to 0.

#### 3.2.3 Self-attention module based on inter-modality and intra-modality collaborations

In the original M4C-VQA model, the characteristic *Z* obtained from multi-modality fusion is input to a Transformer encoder composed of *L* stacked self-attention modules. Each module consists of a multi-head attention (MHA) module and a feed-forward network (FFN) module. MHA combines the outputs of *h* parallel self-attention functions to form diverse characteristic representations by attention fusion. Specifically, the *j*-th self-attention function is defined as:

$$head_{j}(Z)=SA(line_{d_{h}}(Z),line_{d_{h}}(Z),line_{d_{h}}(Z))$$ (5)

$$SA(Q,K,V)=soft\max(QK^{T}/\sqrt{d})V$$ (6)

In which, *d*_*h*_ *= d/h* is the output characteristic dimension of each attention function, and *softma*x represents the softmax activation function.

In contrast, we apply a spatial locational relation modeling method in the INT2-VQA model to modify the self-attention function in (5) to a knowledge priori-guided self-attention function. Specifically, the locational relation knowledge manifestation matrix *R* is obtained by combining *R*^*loc*^ and *R*^*con-sem*^. To ensure that *R*^*loc*^ and *R*^*con-sem*^ have similar value ranges, *R*^*loc*^ is transformed as *R*^*loc*^ = log(*R*^*loc*^ + *ε*), where *ε = 10^(-6)* is a preset constant to prevent numerical underflow during computations. The proposed knowledge-reinforced self-attention function basd on the inter-modality and intra-modality self-attention module, i.e., INT2-SA is defined as:

$$INT2-SA(Q,K,V,R)=soft\max(QK^{T}/\sqrt{d}+R)V$$ (7)

In which, *Q*, *K*, and *V* correspond to the characteristics of *P = K+M+N+T* multi-modality concatenated characteristics *Z*. To achieve knowledge fusion in (7), the knowledge representation matrix *R*∈**R**^*P*×*P*^ is constructed, arranging *R*^*loc*^ and *R*^*con-sem*^ as shown in Fig 6, and filling the remaining positions in *R* with zeros. It should be noted that the usage of *R*^*loc*^ and *R*^*con-sem*^ in INT2-SA is flexible, supporting both inputs simultaneously or any one of the knowledge inputs. Additionally, when *R* is filled with all zero elements, INT2-SA degenerates into a standard self-attention module, corresponding to the standard M4C-VQA model. By combining INT2-SA with the multi-head attention mechanism in (5), the knowledge-reinforced multi-head attention module based on inter-modality and intra-modality collaborations, i.e., INT2-MHA is obtained as:

$$INT2-MHA(Z)=line_{d}([head_{1}(Z),head_{2}(Z),\ldots ,head_{h}(Z)])$$ (8)

#### 3.2.4 INT2-VQA backbone network

By replacing the MHA module in the original M4C-VQA model with the knowledge-reinforced INT2-MHA module, the backbone model of INT2-M4C consists of an *L*-layer depth structure. Specifically:

$${\hat{Z}}^{l}=norm(INT2-MHA(Z^{l-1})+Z^{l-1}),l=1,2,\ldots ,L$$ (9)

$$Z^{l}=norm(FFN({\hat{Z}}^{l})+{\hat{Z}}^{l}),l=1,2,\ldots ,L$$ (10)

In which, *Z*^*0*^ *= Z*, *norm(*.*)* represents layer normalization, *FFN(*.*)* denotes a feed-forward neural network consisting of two layers. The output characteristic *Z*^*l*^ is fed into the auto-regressive answer prediction module in M4C-VQA for iterative answer prediction.

## 4. Experiments and results

To validate the effectiveness of proposed INT2-VQA method, we conducted extensive experiments on the TextVQA dataset, ST-VQA dataset and OK-VQA dataset, which are used on the ST-VQA tasks. We compared the results of INT2-VQA with existing SOTA models and analyzed visually the generated samples from the model.

### 4.1 Dataset and evaluation metrics

The TextVQA dataset consists of 28,408 natural scenario images collected from the Open-Images v3 dataset [48]. The dataset includes 21,953 training images, 3,166 validation images, and 3,289 testing images. For each image, 12 questions are proposed, resulting in a total of 45,336 questions. The training set contains 34,602 questions, the validation set contains 5,000 questions, and the testing set contains 5,734 questions. Each question has 10 manually annotated answers, and accuracy is calculated based on the weighted voting scores of the 10 answers. Following previous work, the top 5,000 frequent words from the training set answers are collected to form a fixed vocabulary.

The ST-VQA dataset [49] includes six datasets: MS COCO, VizWiz, IC-DAR, ImageNet, Visual Genome, and IIIT-STR. It consists of 21,892 natural scenario images, with 18,921 images in the training and validation sets, and 2,971 images in the testing set. For each image, 13 questions are designed, and each question has 12 manually annotated answers. The dataset involves three tasks, and our work follows previous work to evaluate the model on task-3, which is similar to the TextVQA dataset. The official evaluation metric for ST-VQA dataset is the ANLS (average normalized Levenshtein similarity) score for answer consistency assessment. Additionally, the accuracy metric used in the TextVQA dataset is employed as a supplementary evaluation metric in the experiments.

The OK-VQA [50] dataset consists of 14,055 questions, including 9,009 training questions and 5,046 testing questions. These questions are collected from human answers using the Amazon MTurk platform and are composed of image data extracted from the COCO dataset. In order to make the VQA task more human-like and increase the demand for reasoning ability in models, OK-VQA dataset incorporates external knowledge resources. As a result, some questions require leveraging external knowledge to answer. The evaluation metric used for this dataset is VQA-score, which combines accuracy and program consistency accuracy, considering the scope of external knowledge on which the model relies when answering questions. The dataset split follows [50], simply dividing it into a training set with a sample size of 9,009 and a testing set with a sample size of 5,046.

### 4.2 Experimental setup

The experiments were conducted on a workstation equipped with an Nvidia Titan X GPU. The proposed INT2-VQA model was implemented using the Tensorflow framework. Following previous work, the hyper-parameters for model design and training are as follows: the maximum number of words in a question, K, is 20; the maximum number of target entities, M, is 100; the maximum number of OCR targets, N, is 50; the maximum length of an answer, T, is 12; the unified representation dimension, d, is 768; the number of attention heads, h, is 12; the vocabulary size, V, is 5,000; the optimizer used is Adam; the batch size is 128; the base learning rate is 10^(-4); the learning rate decay factor is 0.1; the learning rate decay steps are 14,000/19,000; and the number of iterations is 24,000.

### 4.3 Ablation experiments

To evaluate the effectiveness of different model architectures compare to proposed INT2-VQA, ablation experiments were conducted on the TextVQA dataset. For a fair comparison, all models in the ablation experiments used Microsoft OCR to extract textual information from images and employed a Faster R-CNN model with a ResNeXt-152 backbone for visual characteristic manifestation [51]. The experimental results are shown in Table 1.

**Table 1:** Ablation results.

Methods	Model layers (Structure)	Validation set accuracy/%
M4C-VQA	6 (N)	45.75
SA-M4C-VQA	6 (S)	46.83
**INT2-VQA**	**6 (K)**	**48.92**
INT2-VQA(remove *R*^*lo*c^)	6 (K)	46.90
INT2-VQA(remove *R*^*con-sem*^)	6 (K)	47.06
SA-M4C-VQA	2 (N) -> 4 (S)	46.70
INT2-VQA	2 (N) -> 4 (K)	47.91
INT2-VQA	3 (N) -> 3 (K)	47.04
INT2-VQA	4 (N) -> 2 (K)	47.18
INT2-VQA	4 (K)	46.10
INT2-VQA	8 (K)	48.34
INT2-VQA	10 (K)	48.31

Note: Bold font indicates the best results, and the letters in parentheses represent different attention structures: N denotes the standard attention structure, S denotes the spatial-aware attention structure in SA-M4C-VQA, and K denotes the proposed attention structure with knowledge representation enhancement, i.e., INT2-SA.

#### 4.3.1 Enhancement effect of different types of knowledge

Compared with the M4C-VQA model architecture, the enhancement effect of introducing different types of knowledge was verified. First, comparing the results in rows 1 and 3 of Table 1, it is observed that under the same number of layers (both 6 layers), the proposed INT2-VQA method significantly outperforms the M4C-VQA method, demonstrating the effectiveness of introducing knowledge manifestation enhancement based on the inter-modality and intra-modality collaborations. Second, comparing the results in rows 1, 3, 4, and 5, it is found that methods that only model locational collaboration knowledge Rloc or contextual semantical collaboration knowledge Rcon-sem both lead to a certain degree of performance degradation, but still show improvement compared to the M4C-VQA method, demonstrating that both types of knowledge contribute to the model to some extent, and they have complementary characteristics. Finally, comparing the results in rows 2 and 5, although SA-M4C-VQA and INT2-VQA with only locational collaboration Rloc employ similar strategies for knowledge manifestation enhancement, the locational correlation modeling granularity in M4C-VQA is coarser than that in INT2-VQA, resulting in slightly lower accuracy compared to the proposed INT2-VQA method.

#### 4.3.2 Influence of different module combinations

In M4C-VQA model, combining different types of attention modules, such as two standard self-attentions (N) in M4C-VQA and four spatial-aware attention modules (S) in SA-M4C-VQA, can enhance the model’s expressive power. Therefore, in the results of rows 3, 8–11 in Table 1, with the number of layers L = 6 fixed, the impact of different combinations of attention modules, namely the standard attention module (N) in M4C-VQA and the knowledge manifestation enhancement attention module based on the inter-modality and intra-modality collaborations (K) in INT2-VQA, was explored. The 6(K) architecture, which incorporates knowledge, achieved the best results in each layer. Compared to the a (N) -> (6-a) (K) architecture in rows 7–9, the accuracy was improved by at least 0.5%. However, this result is inconsistent with the results in the M4C-VQA of original paper. The reason might be that the locational information encoding strategy in M4C-VQA imposes strong constraints, which weaken the comprehension of the contextual semantical information in the early layers. In contrast, INT2-VQA introduces a set of independent learnable parameters when fusing knowledge at each layer, enabling the model to adaptively learn the degree of knowledge fusion at different layers and achieve better fusion effects.

#### 4.3.3 Influence of different stacking depths

Keeping the architecture design of L(K) constant, the performance of the model was explored with different depths L. From the results in rows 3, 10, and 11 of Table 1, it can be observed that as the depth L increases from 4 to 10, the performance of the proposed INT2-VQA model initially improves and then gradually decreases. The optimal result is achieved when L = 6. The reason for this phenomenon is that overly deep models can lead to optimization difficulties and limit the model’s expressive power. This problem may be alleviated by introducing more training data.

### 4.4 Contrast experiment

Based on the results of the ablation experiments, we select INT2-VQA method with 6(K) architecture comparing with other SOTA methods in this section. The compared methods include LoRRA, MM-GNN [52], M4C-VQA, SMA [53], CRN [54], LaAPNet [55], and SA-M4C-VQA. The results are shown in Table 2.

**Table 2:** Comparative results based on the TextVQA.

Methods	Backbone	OCR	Additional training data	Validation set accuracy /%	Testing set accuracy /%
LoRRA	ResNet	Rosetta-ml	N/A	26.56	27.63
MM-GNN	ResNet	Rosetta-ml	N/A	31.44	31.10
M4C-VQA	ResNet	Rosetta-en	N/A	39.40	39.01
SMA	ResNet	Rosetta-en	N/A	40.05	40.66
CRN	ResNet	Rosetta-en	N/A	40.39	40.96
LaAPNet	ResNet	Rosetta-en	N/A	40.68	40.54
INT2-VQA (our)	ResNet	Rosetta-en	N/A	41.78	42.99
M4C-VQA	ResNet	Rosetta-en	ST-VQA	40.55	40.46
LaAPNet	ResNet	Rosetta-en	ST-VQA	41.02	41.41
INT2-VQA (our)	ResNet	Rosetta-en	ST-VQA	42.78	43.51
SA-M4C-VQA	ResNeXt	Google OCR	N/A	43.90	N/A
SA-M4C-VQA	ResNeXt	Microsoft OCR	N/A	46.70	N/A
INT2-VQA (our)	ResNeXt	Microsoft OCR	N/A	48.42	N/A
SA-M4C-VQA	ResNeXt	Microsoft OCR	ST-VQA	47.99	48.52
**INT2-VQA (our)**	**ResNeXt**	**Microsoft OCR**	**ST-VQA**	**49.27**	**49.63**

Note: Bold font is the optimal result of different comparison conditions. N/A indicates no experimental data. Additional training data refers to data other than the TextVQA dataset, such as the ST-VQA dataset.

Table 2 shows a fair comparison of different conditions, such as characteristic extraction backbone, OCR system, and additional training data. The characteristic extraction backbone includes a combination of Faster R-CNN with ResNet and ResNeXt backbones. The OCR system includes the OCR systems used by each method. Most methods adopt the Rosetta-ml and Rosetta-en systems provided by Facebook, while SA-M4C-VQA uses a better-performing Google OCR system. This study uses the Microsoft OCR system.

From the experimental results, the following conclusions can be drawn:

1. Under the conditions of using the Rosetta-en OCR system and Faster R-CNN with ResNet-101 backbone, the proposed INT2-VQA achieves accuracies of 41.78% and 42.99% on the validation set and testing set, respectively (row 7), with improvements of 1.1% and 2.5% compared to the best results.
2. Adding the ST-VQA dataset as additional training data, INT2-VQA achieves accuracies of 42.78% and 43.51% on the validation set and testing set, respectively (row 10), which are the best results under the same conditions.
3. Replacing the Google OCR system with the better-performing Microsoft OCR system without adding additional data, the INT2-VQA model achieves a 1.7% improvement in accuracy on the validation set compared to the SA-M4C-VQA model under the same conditions (row 13).
4. Adding the ST-VQA dataset as training data, the INT2-VQA model achieves the best accuracies of 49.27% and 49.63% on the validation set and testing set, respectively, surpassing SA-M4C-VQA by 1.3% and 1.1% under the same conditions.

To validate the effectiveness of the proposed INT2-VQA method, contrast experiments were also conducted on the ST-VQA dataset, and the results are shown in Table 3.

**Table 3:** Comparative results based on the ST-VQA.

Methods	Validation set accuracy/%	Validation set ANLS	Testing set ANLS
SAN & STR	N/A	N/A	0.135
M4C-VQA	38.05	0.472	0.462
SMA	N/A	N/A	0.466
CRN	N/A	N/A	0.483
LaAPNet	39.74	0.497	0.485
SA-M4C-VQA	42.23	0.512	0.504
**INT2-VQA**	**46.99**	**0.562**	**0.555**

It can be observed that the proposed INT2-VQA method outperforms the existing best result by 4.7% in accuracy on the validation set and improves the ANLS metric by 5% on both the validation set and testing set.

In order to further validate the generalization of our proposed method, we conducted comparative experiments on the OK-VQA dataset with the latest methods, including DSGEM (Dual Scene Graph Enhancement Module), OECA-Net, and MAGM (multimodal adaptive gated mechanism model). The results following the best settings in the original paper are shown in Table 4. Here is an introduction to these methods:

**Table 4:** Comparative results based on the OK-VQA.

Methods	Testing set VQA score
OECA-Net	26.78
DSGEM	27.93
MAGM	35.46
INT2-VQA	41.77

DSGEM [56] utilizes commonsense knowledge and syntactic structures to construct visual and textual scene graphs, explicitly assigning specific semantics to each edge relation. Two scene graph enhancement modules are proposed to propagate external and structural knowledge, providing clear guidance for feature interactions between objects. Finally, these two scene graph enhancement modules are embedded into the existing VQA model, introducing explicit relation reasoning capability.

OECA-Net [57] designs a model based on co-attention mechanisms, including question self-attention unit, question-guided image visual attention unit, and question-guided image OCR token attention unit. The question self-attention module filters redundant question information. The question-guided attention modules are used to obtain the final visual features and OCR token features from the image. The question-guided image OCR token attention unit fuses the question text features, visual image features, and OCR token features from the image.

MAGM [58] introduces an adaptive gating mechanism in the process of intra-modal, inter-modal learning, and modal fusion. This model can effectively filter out irrelevant noise information, obtain fine-grained modal features, and improve the model’s adaptive control over the contribution of two modal features to answer prediction. In the intra-modal and inter-modal learning modules, self-attention gating and self-guided attention gating units are designed to effectively filter noise information from text and image features. In the modal fusion module, an adaptive gating modal feature fusion structure is designed to obtain fine-grained modal features, thereby improving the accuracy of the model’s question answering.

The OK-VQA dataset is considered effective in assessing a model’s ability to reason about unknown information and its ability to tackle challenging questions. The results in Table 4 demonstrate that, despite the relatively low performance of baseline methods, the proposed method still outperforms them significantly.

In addition, a comparison of the model sizes and complexities of M4C-VQA, SA-M4C-VQA, and INT2-VQA was conducted. The results are shown in Table 5.

**Table 5:** Comparative results about model sizes and complexities.

Methods	Parameter quantity/ M	FLOPs/G	Inference time/ms
M4C-VQA	96.63	18.3	89
SA-M4C-VQA	96.63	18.4	118
INT2-VQA	96.65	18.4	132

It can be observed that the 6(K) architecture of INT2-VQA has almost no increase in model parameters and FLOPs compared to the 6(N) architecture of M4C-VQA and the 2(N) + 4(S) architecture of SA-M4C-VQA. The additional inference time of INT2-VQA compared to M4C-VQA and SA-M4C-VQA is mainly used for computing the cosine similarity between predicted answer words and OCR words. Optimizing this computation process to further improve the computational efficiency of the method is an important future work.

### 4.5 Visual analysis

**Fig 7:**
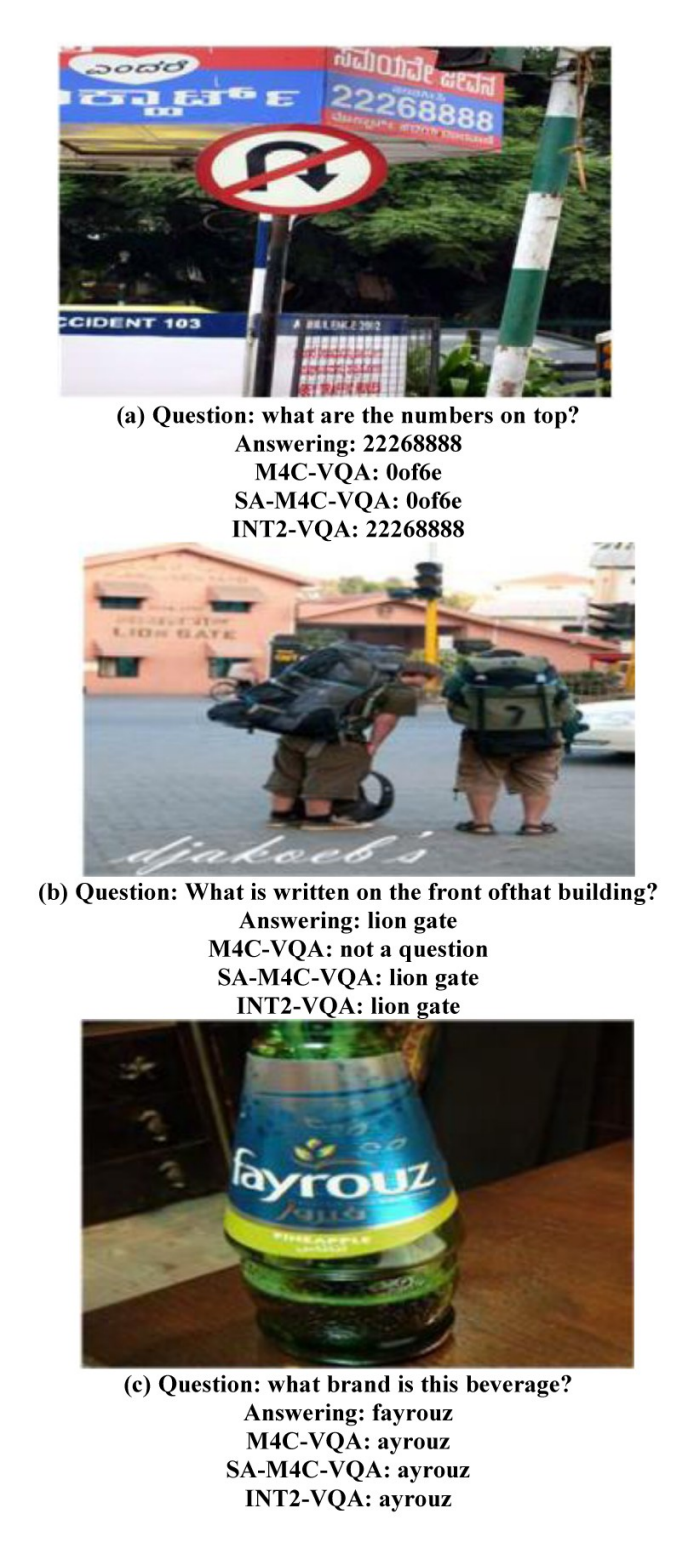
Visual analysis of typical scenarios. **(a)** Question: what are the numbers on top? Answering: 22268888. M4C-VQA: 0of6e. SA-M4C-VQA: 0of6e. INT2-VQA: 22268888. **(b)** Question: What is written on the front of that building? Answering: lion gate. M4C-VQA: not a question. SA-M4C-VQA: lion gate. INT2-VQA: lion gate. **(c)** Question: what brand is this beverage? Answering: fayrouz. M4C-VQA: ayrouz. SA-M4C-VQA: ayrouz. INT2-VQA: ayrouz.

To better understand the performance of INT2-VQA, several typical examples were selected for analysis, and the relevant results are shown in Fig 7.

It can be observed that:

1. INT2-VQA achieves better results compared to M4C-VQA and SA-M4C-VQA, demonstrating the improvement in the model’s ability to comprehend scenario text with the introduction of knowledge enhancement based on the inter-modality and intra-modality collaborations.
2. SA-M4C-VQA and INT2-VQA show significant advantages over M4C-VQA when it comes to relative spatial and locational relations. They can accurately answer complex spatial reasoning tasks.
3. Benefiting from the contextual semantical collaboration knowledge established by INT2-VQA, the proposed model can discover the implicit semantical collaborations between predicted answer words and OCR words. Therefore, it performs better when dealing with answers involving multiple words compared to SA-M4C-VQA, which only considers spatial relations.
4. When faced with complex scenarios that require associative abilities to comprehend partial scenario information, all methods perform poorly. This reflects the performance bottleneck of existing ST-VQA frameworks and calls for further in-depth research.

## 5. Discussion

At the current stage, VQA tasks are still in need of continuous development and further research. There are still many problems and challenges in different types of tasks, such as improving task functionality and characteristic integration. This section combines existing VQA methods to provide insights into potential future research directions.

More general pretraining methods. Models based on the Transformer framework have made significant advancements in various fields such as natural language processing and computer vision. This suggests the possibility of using a unified Transformer architecture to learn multi-modality knowledge for different tasks in different domains. However, existing models often require fine-tuning when applied to downstream tasks to achieve significant performance improvements. Currently, there are some general pretraining models [59] that can be applied to multi-modality tasks such as VQA. For example, literature [60] proposes a unified model pretraining architecture that improves textual and visual comprehension capabilities by leveraging large-scale free-text corpora and image collections, aligning textual and visual information in a unified semantical space through cross-modal contrastive learning. Although this pretraining model achieves generality in cross-modal tasks, further research is needed to explore universal pretraining methods that can be applied to purely visual tasks, purely textual tasks, as well as visual-textual cross-modal tasks without the need for fine-tuning. Additionally, researchers can explore large-scale pretraining datasets with more complex and diverse types of knowledge to learn more generalized characteristics.

Generative visual question answering methods. Currently, most VQA tasks are defined as the classification problems. However, in practical applications, there can be multiple correct answers to a question. Therefore, classification-based VQA systems to some extent limit the model’s development. Thus, depending on different scenarios and questions, VQA systems should have the ability to directly provide accurate and precise answers. Although there are some generative VQA tasks, such as a literature [61] that proposes a framework for generative VQA, which takes images and questions as input and generates a reasonable and accurate answer with the assistance of effective annotations from human annotators and network data noise. Since generative question-answering tasks have just emerged in the field of VQA, the answers generated by existing methods are not precise enough and often rely only on surface-level information from visual images, lacking the integration of deep-level information such as commonsense knowledge. Therefore, constructing datasets with richer varieties of image-question pairs can enable models to generate more diverse answers. Moreover, the generative setting is conducive to the transfer of models to other tasks such as visual dialogue. Therefore, the generative setting offers broad research opportunities that are worth exploring further.

Construction of novel VQA datasets and evaluation metrics. Currently, the datasets used for evaluating and testing various tasks are not well-balanced, and they contain various biases. The evaluation metric for models are relatively rough, especially for open-ended VQA tasks, where specific standards and improvement measures need to be proposed for evaluating the accuracy, coverage, and fusion of cross-modal characteristics. Additionally, there are challenges in determining evaluation metric for generative image question-answering models, such as the difficulty in generating answers and evaluating them. Specifically, the fusion level between different modal characteristics and the reasoning process of model answer generation require the establishment of more intuitive and visual evaluation systems. There is an urgent need for in-depth research and standardization of specialized datasets and data types for improving VQA in different domains and addressing its shortcomings.

## 6. Conclusion

We propose a scenario text-based VQA method that integrates knowledge manifestation based on inter-modality and intra-modality collaborations. Building upon the baseline method M4C-VQA, we introduce two complementary priori knowledge sources, i.e., locational collaboration between modalities and contextual semantical collaboration within modalities. We introduce the INT2-VQA model, which enhances knowledge manifestation, to achieve unified modeling and expression of both knowledge sources and multi-modality data. We validate our approach on two commonly used scenario text-based VQA datasets and achieve significant performance improvements compared to existing SOTA methods. The proposed framework is not only applicable to scenario text-based visual tasks but also provides a platform for improving other related multi-modality learning tasks.
